# Can telemedicine help integrate the referral-based healthcare system? An economic and operational analysis

**DOI:** 10.1371/journal.pone.0336490

**Published:** 2025-11-14

**Authors:** Xuejing Cao, Guohao Li, Heng Zhao

**Affiliations:** 1 School of Business, Shaoxing University, Shaoxing, China; 2 School of Economics and Management, Tongji University, Shanghai, China; Universitatsklinikum Schleswig Holstein Campus Lubeck, GERMANY

## Abstract

**Purpose:**

Telemedicine can help specialists in providing efficient care to patients from a distance and it has been growing steadily in healthcare. This study aims to provide an operational perspective by investigating the effect of telemedicine on patients’ choices, healthcare providers’ service decisions, service quality and total social welfare.

**Methods:**

We propose an optimization model that incorporates patients’ choices and physicians’ actions under two scenarios: full coverage and partial coverage. We analyze the model and conduct numerical experiments to explore the impact of telemedicine in a referral-based healthcare system with heterogeneous patients.

**Results:**

The findings show that telemedicine can induce greater collaboration between generalists and the specialist, which would result in the specialist spending less time on each patient. Interestingly, we find that after the introduction of telemedicine, the average quality cost decreases under full coverage but increases under partial coverage. Moreover, the introduction of telemedicine could lead to higher social welfare as it improves the accessibility of services to patients, especially in rural areas. Finally, this study also demonstrates that the referral-based healthcare system may benefit more from telemedicine when there is a heavier travel burden for patients or a higher financial incentive for generalists.

**Conclusion:**

The introduction of telemedicine mainly contributes to the patients’ service accessibility, particularly for patients in remote areas, and can foster closer collaboration between generalists and specialists. However, in hospitals where medical resources are already strained, it may worsen specialist overuse and lead to lower service quality. These findings highlight that telemedicine is not universally beneficial. The policymaker should consider regional coverage conditions and use targeted financial incentives to improve its effectiveness within referral-based healthcare systems.

## 1. Introduction

There are two parts in a referral-based hierarchical healthcare system: primary care providers (equivalent to generalists, general practitioners) and specialty care from hospitals [1,2]. Generalists play the role of gatekeepers and care coordinators, deciding whether to treat the patients or refer them to hospitals dedicated to specialty care which has high-quality medical resources [3]. However, patients’ health-seeking behavior may differ in the practice of hierarchical healthcare systems. In settings similar to the NHS in the United Kingdom and the HMO in the United States, patients usually see a generalist for an initial consultation, whereas patients in China usually prefer to see specialists directly since primary care providers are not trusted [4].

However, the specialty care delivery may be constrained by the regional health resource disparities [5]. In rural or remote areas, patients face significant barriers to accessing specialist services, primarily due to high travel costs and long distances. Dorsey et al (2013) showed that it commonly takes patients from remote or rural areas four hours to visit a specialist but only have a half-hour meeting there [6]. Travel cost includes but is not limited to time cost and extra financial cost, besides, patients with limited mobility (e.g., elderly patients) may incur the risk of worsening condition during the long journey. Therefore, generalists may take into account the travel cost when making the referral decision, and the patients may evaluate the cost-effectiveness of referral likewise, determining whether to follow up the decision [7–9]. Earlier studies have suggested that longer distances/travel-time are associated with lower referral rate [10–12].

These access-related challenges are further exacerbated by broader systemic pressures. The global healthcare system is confronting a combination of aging populations, rising prevalence of chronic diseases, and growing demand for equitable and efficient care. In large countries like China, such issues are particularly acute due to the geographic imbalance in provider availability and institutional capacity. In response to these challenges, telemedicine has emerged as a promising solution to bridge gaps in access, improve care coordination, and reduce patient burden in hierarchical referral-based healthcare systems. Governments and healthcare systems around the world are actively promoting the adoption of telemedicine as part of broader digital health transformation efforts.

Telemedicine is a subset of the broader concept of telehealth, which encompasses not only clinical services but also administrative activities, provider training, and continuing medical education. In contrast, telemedicine refers specifically to the remote provision of clinical services using information and communication technologies. It enables face-to-face communication between highly trained doctors and patients who are not convenient to go to hospitals to seek treatment, with the goal of improving the efficiency of healthcare delivery [9,13], mitigating health disparities [14,15], and reducing the risk of exposure and cluster infection during the pandemic [16,17]. The concept of telemedicine in our study is similar as the technology referred in Sun et al (2020)’s work, telemedicine help providers treat patients remotely by establishing a medical resource-sharing network and on-offline collaboration, and patients will be treated by the SP with the assistance of the GP after the referral [18]. The policymaker encourage the service capacity sharing among institutions or providers since it could improve access to services [19], therefore they generally implement a bundle payment scheme for each episode of care to promote the telemedicine application, which is viewed as a new tool for enhancing the coordination and integration of the healthcare providers.

In the field of operations management, a growing number of analytical studies employ mathematical modeling approaches to investigate problems related to telemedicine. For example, Rajan et al. (2019) demonstrated that the introduction of telemedicine enhances specialist diagnostic efficiency and increases overall social welfare, yet it may leave some patients with diminished benefits [20]. Çakıcı and Mills (2021) specifically analyzed teletriage systems, highlighting their potential operational risks in healthcare delivery [17]. They noted that patients facing high diagnostic uncertainty could achieve better cost outcomes through teletriage, whereas high overtriage rates paradoxically increase emergency department (ED) visitation frequency. Guan et al. (2025) revealed that teletriage may prompt patients with milder conditions to seek care while deterring those with more severe cases [21]. This effect occurs when teletriage systems lack sufficient precision and when patients’ treatment-seeking hassle costs deviate from moderate levels. Such findings caution the policymaker that teletriage might exacerbate rather than alleviate healthcare inefficiencies [22].

Some studies have similarly examined how telemedicine affects referral patterns within healthcare systems. Wang et al. (2023) indicated that smaller hospitals are better positioned to use telemedicine as a triage tool for patient screening, whereas larger hospitals tend to view it as a professional medical service channel for patient care. Their study demonstrates that under partial market coverage, total social welfare consistently exceeds pre-implementation levels, though profit outcomes remain uncertain. However, in fully covered markets, both profitability and social welfare invariably decline compared to pre-adoption baselines. In contrast, our research focuses on a more narrowly defined hierarchical healthcare system, where patient referrals are coordinated across different tiers of hospitals rather than between online and offline service channels. Moreover, in our research framework, only large hospitals are assumed to effectively utilize telemedicine.

The legal and technical boundaries of telemedicine vary by country but generally require healthcare providers to adhere to licensing regulations, protect patient data privacy, and ensure the reliability of digital communication platforms. Institutionally, telemedicine in China is guided by the National Health Commission’s digital health agenda, which focus on inter-hospital collaboration and reimbursement under bundled payment schemes. Telemedicine provides prospects for expanding specialist access, lowering patient travel burdens, and improving care coordination. However, challenges remain, including uneven digital infrastructure across regions, limited interoperability of electronic health records, regulatory ambiguity in cross-provincial service delivery, and the risk of quality dilution owing to an overreliance on remote consultation. These realities highlight the importance of developing talored implementation strategies across healthcare settings.

In this study, we first construct a stylized model and then examine the impact of telemedicine on the referral process, service delivery, and social welfare within a hierarchical healthcare system. The system is simplified to include a specialist provider, nearby general practitioners and spatially distributed patients. A key feature of the referral-based healthcare system is the visibility and attractiveness of different hospitals. For example, top-tier tertiary hospitals in metropolitan areas such as Beijing and Shanghai attract patients nationwide due to their reputation and advanced capabilities. If some patients in such a specialist-centered healthcare delivery system decide not to seek specialty care due to prohibitively high travel costs, we define the system as **partial coverage**, which is more likely to exist in tertiary hospitals in practice. In contrast, ordinary hospitals often face lower demand and have sufficient capacity to fully accommodate patient needs within their local catchment areas, resulting in a **full coverage** scenario. This distinction motivates our comparison of telemedicine’s effectiveness across the two scenarios.

Following the seminal paper in referral-based service system [23], we incorporate a telemedicine component wherein SPs can treat patients with remote assistance from GPs via telemedicine. We treat patient populations as a heterogeneous group in terms of distance from the specialist, as inspired by Rajan et al (2019) [20]. Each patient chooses between GP and SP to maximize expected utility, while the GP optimizes referral levels under incentive constraints, and the SP adjusts service rate to maximize profit.

We investigate the impact of telemedicine on providers’ service decisions, service quality and total social welfare. We seek answers to the following operational details: How will patients in a referral-based healthcare system react to telemedicine implementation? How will the GP and SP react to the introduction of telemedicine? How do the aforementioned reactions affect service delivery within a referral-based healthcare system? Lastly, when and where will telemedicine become economically viable? Our results provide some insights with practical implications, which are of guidance to the policymaker.

Our main contributions are fourfold: (i) we model patient heterogeneity in spatial access and compliance behavior; (ii) we distinguish between full and partial coverage regimes; (iii) we incorporate bundled payment incentives for GPs under telemedicine. While prior studies have examined these elements separately, we provide a unified framework to understand the joint dynamics of patient behavior, provider incentives, and systemic outcomes.

The remain of this study is organized as follows. We thoroughly describe the problem and the characteristics of patients and service providers in section 2. In section 3, we formulate the patient’s utility function, GPs’ objective function and SP’s objective function before and after the introduction of telemedicine. In section 4, we provide the results of numerical experiments performed to supplement the analytical results. We discuss the implications of our results for healthcare policy in section 5.

## 2. Problem description

To ground our analysis, we consider institutional features that reflect the healthcare context in developing countries, using China as a motivating example. Healthcare is delivered through a hierarchical system, where patients are encouraged to seek primary care from general practitioners (GPs) before being referred to specialists (SPs). However, in practice, patients often bypass primary care due to perceived quality gaps. Although patients formally have freedom of provider choice, their actual decisions are shaped by travel costs, waiting times, and trust in primary care. Referrals by GPs are encouraged but not mandatory. Compliance with referrals is affected by patients’ perception of the cost-effectiveness of treatment paths. The government acts as a social planner, reimbursing patients for a portion of their medical costs as well as channeling medical services to providers. However, regional disparities in telemedicine adoption and reimbursement policy persist. These features inform the assumptions in our model regarding patient cost sensitivity, referral incentives, and the role of tele-rewards.

We mainly analyze the impact of telemedicine on patient’s choice, provider’s actions, and social welfare in this referral-based system. In addition, it should be noted that we consider a number of GPs and a single SP in the setting. Patients can visit both the nearest GP or the SP based on their expected utility, and providers cannot refuse to provide services and treatments to patients.

We assume that patients with complexity x∈[0,1] uniformly distributed in $d\in [\text{0},d_{max}]$, where $d_{max}$ is the maximum service range of the SP and the population of patients served by the insurer is exogenous and, without loss of generality, equal to $d_{max}$. Appendix A in S1 Appendix summarizes the notations for all the variables and parameters in this study.

We assume that all GPs have the same diagnostic capabilities. Following Shumsky and Pinker (2003) [23], let k∈[0,1] be GPs’ referral level such that patients with complexity x≤k are treated by GPs. Let f(x) denote the probability that GP can successfully treat the illness, given that the patient’s severity of illness is x. From the definition of k, $f^{\prime }(k)<\text{0}$. It implies that the probability that GP can successfully cured patients with a pretty low complexity is high enough and vice versa. Let F(k) be the expected fraction of all patients that are successfully treated by GP, given that GPs’ referral level is k, so that $F(k)\equiv \int_{\text{0}}^{k}f(x)dx$.

When a patient visits a GP, the GP would make an initial diagnosis and decide whether or not refer her to SP, depending on the severity or complexity of the patient’s illness. Therefore, the total proportion of patients referred to SP is 1−F(k). Before the introduction of telemedicine, patients who were referred could only go directly to SP to register and wait in line for treatment. In this case, when the patients are far away from the hospital, they may care about the high distance cost and give up treatment. After the introduction of telemedicine service, patients who are not capable of being treated by GP can be treated remotely by SP with the assistance of GP. In this case, the providers share a single bundled payment. We denote γ as GP’s tele-reward when he assists in the telemedicine. Associated with each patient not treated successfully by the GP, and subsequently referred to a SP, is a mistreatment cost m. This is a cost suffered by the patient due to misdiagnosis and treatment failure and may include medications, scans, hospitalization, etc. The main causes of medical disputes in health care services are the technical reasons of community hospitals, which are manifested by missed diagnosis, misdiagnosis, and improper medication. Medical disputes not only affect patients, but also cause community hospitals to lose credibility and receive penalties from the government. Therefore, GPs should not only consider costs incurred during treatment but also take mistreatment cost into account in his objective function in making decisions. Considering that GPs are widely distributed and accessible in real life, we assume that there are a number of GPs are distributed in a region and treat them as a whole, without considering the distance and queuing problems between patients and GPs. We consider a model with no capacity constraints or congestion at GPs.

We assume that there is only one SP serving a group of patients in a region. SP treats patients that choose to go to SP for her first treatment and that are referred to him by the GP, either after diagnosis or after unsuccessful treatment. let $\lambda_{s}$ represent the arrival rate at the SP. SP decides his service rate μ for the purpose of maximizing profits. Service times follow an exponential distribution with mean $\frac{\text{1}}{\mu }$. Service rate μ reflects the average service effort or treatment intensity devoted to a patient. $\frac{C_{s}}{\mu }$ is the service cost at SP to treat a patient, in which $C_{s}$ is the service cost of SP per unit time. Following Rajagopalan and Tong (2022)’s work, αμ is denoted as the cost of inadequate care, such as the recurrence of the patient’s disease due to incomplete treatment [24]. It is obvious that αμ is an increasing function of μ. However, a long service time also leads to more congestion in queues, which can exacerbate patients’ symptoms. Barua et al (2014) found a link between long waiting time for outpatient care and negative health outcomes such as morbidity, mortality or readmission rates [25]. We denote $\beta W(\lambda_{s},\mu )$ as patient’s expected waiting cost incurred at SP, where β represent delay care cost per unit time and $W(\lambda_{s},\mu )$ is average waiting time per patient.

For simplicity, we assume that there is no difference in the hospital’s service rate μ for patients with different severity of disease and patients are gonna end up being cured. In addition, after the introduction of telemedicine, SP would not make a difference between patients go to SP in person and patients who choose telemedicine in terms of service rate and treatment price. We model the congestion at the SP $W\left(\lambda_{s},\mu \right)=\frac{\text{1}}{\mu -\lambda_{s}}$ using an *M/M/1* queueing model. $\pi_{s}$ is denoted as SP’s profit.

### 2.1. Assumption 1. $\frac{\partial \pi_{s}}{\partial \lambda_{s}}>0$

Assumption 1 implies that SP’s profit increases in patients’ arrival rate. Thus, the SP aims to maximize his arrival rate given any GPs’ referral level.

The government has offered financial incentives to encourage patients to visit GPs first, however, due to the limitation of GPs’ ability to treat complex patients, most of patients still want to visit the SP directly. Given GPs’ referral rate and SP’s service rate, patients would make a decision according to their expected utility. The patient’s expected utility consists of five components:

(i) The reward $R_{i},i\in \{g,s,t\}$ from seeking healthcare service, where $R_{g},R_{s},R_{t}$ represents the reward of a patient when she visits a GP, the SP in person, and telemedicine with the assistance of a GP respectively. Beacause of the weakness of patients’ perception of their current health status, for simplicity, we assume that each patient gets a constant reward $R_{i}$ when she is cured. Patient’s reward is not only related to the treatment outcomes, but also related to the experience in the service, such as the physician’s service attitude and the perception of the physician’s professionalism. Generally speaking, the expertise and patient satisfaction of tertiary hospitals are usually higher than that of community hospitals. Meanwhile, patients cannot communicate with SP in person when employing telemedicine with the assistance of GP, that is, patient’s reward from telemedicine is lower than in person visiting. Then, we assume $R_{s}>R_{t}>R_{g}$.
(ii) Treatment expense $\left(\text{1}-q_{i}\right)P_{h},i\in \left\{g,s,t\right\},h\in \{g,s\}$. $P_{g}$ and $P_{s}$ are denoted as the price of service from GPs and SP respectively, where $P_{g}<P_{s}$. We denote $q_{g},q_{s},q_{t}$ as the reimbursement rate of GPs, SP in person, and SP via telemedicine respectively. In developing countries, such as China, the government is pushing the reform of referral-cased healthcare system and integrated healthcare organizations (one form is telemedicine) through offering financial incentives. Then, we let $q_{g}>{q_{t}>q}_{s}$ in this model.
(iii) Travel cost $C_{t}d$. One of the most significant benefits of telemedicine is that it obviates the need to travel for some consultations with the specialist. We use $C_{t}d$ to model patients’ travel cost, where $C_{t}$ is the travel cost per unit distance and d denotes the distance between SP and patient, which is uniformly distributed on the interval from 0 to $d_{max}$. It’s worth noting that we do not consider the patient’s travel cost at GP because of the broad distribution of GPs. This is a crucial point because of our ultimate goal of understanding the effect of telemedicine on patients’ and providers’ choices.
(iv) Mistreatment cost at GP m[k−F(k)]. For the sake of simplicity, we do not consider the differences in health status and personal cognition among patients. When a patient visits a GP, she may be misdiagnosed with the probability of k−F(k).
(v) Quality cost at SP $QC\left(\lambda_{s}\right)=\frac{\beta }{\mu -\lambda_{s}}+\alpha \mu$. We model the service quality cost of patient using waiting time of patient and the service rate of SP. The waiting cost at the SP $\frac{\beta }{\mu -\lambda_{s}}$ is the cost of delayed care due to waiting for the service. αμ is care quality cost which decreases with increased treatment time and this function captures the diminishing marginal effect of increased service time on care quality cost.

The goal of the government is usually to maximize the efficiency and performance of the healthcare system, that is, while providing services to patients, it should also maximize total social welfare, including the reward from seeking healthcare service, treatment costs of GPs and SP, as well as service quality cost.

### 2.2. Remark: Clarifications on modeling assumptions

In our model, patients may suffer mistreatment costs if they receive inappropriate care from GPs who lack the capacity to treat complex cases. These costs capture the potential disutility caused by misdiagnosis, or treatment errors. While the providers themselves are assumed to be profit-oriented and do not explicitly internalize patient disutility (i.e., we do not assume provider altruism), the model structure indirectly incorporates quality trade-offs through mistreatment penalties, which influence both referral incentives and patient routing behavior.

To obtain partial analytical insights, we did not assume that patients’ utility is a general function of case complexity k. Instead, we simply let the utility depend on the service provider and whether a patient gives up going to a tertiary hospital is mainly determined by the queuing time and travel distance.

Regarding the definitions of waiting time and quality-related costs, we acknowledge the overlap and clarify that the quality cost is a broader concept, incorporating the effects of both mistreatment and congestion at the SP. We define the SP’s waiting cost as increasing in service rate due to congestion, and the quality loss as the resulting degradation of care from reduced time per patient.

Different prices and reimbursement rates for providers reflect realistic institutional settings, where GPs and SPs operate under different payment schemes. For example, specialists in hospitals may receive higher fees per service, and insurance may cover different proportions of GP and SP costs depending on policy design. These asymmetries influence provider incentives and patient choice, and are modeled through differentiated service prices and copayments.

## 3. Methods

Based on above settings and assumptions, we construct two analytical models of before and after the introduction of telemedicine respectively.

In this paper, we also assume that patients are rational and strategic when making decisions, then, a mixed strategy Nash Equilibrium will be reached in this setting.

### 3.1. Before the introduction of telemedicine

We first give the patient’s expected utility in a base model where the patient can freely make a decision between going to a GP first and going directly to the SP without telemedicine. We then mainly examine how GPs and the SP make referral decision and service rate decision to maximize their own profits. Let $U_{s}^{b}$ and $U_{g}^{b}$ denote patient’s expected utility for visiting GPs and the SP before the introduction of telemedicine, respectively, which are given by:

(1) Patient’s expected utility function at SP:

$$U_{s}^{b}=R_{s}-\left(\text{1}-q_{s}\right)P_{s}-QC\left(\lambda_{s}^{b}\right)-C_{t}d$$ (1)

(2) Patient’s expected utility function at GP:

$$U_{g}^{b}=F\left(k_{b}\right)R_{g}-k_{b}\left(\text{1}-q_{g}\right)P_{g}-m\left[k_{b}-F\left(k_{b}\right)\right]+\left[\text{1}-F\left(k_{b}\right)\right]U_{s}^{b}$$ (2)

where $k_{b}$ represents GP’s referral rate without introducing of telemedicine.

We next analyze patients’ choices given the above functions. In a two-tier healthcare system, given GPs’ referral rate $k_{b}$, SP’s service rate $\mu_{b}$, and other parameters, a patient makes her decision by comparing the expected utilities from visiting in either service providers. Specifically, she visits a GP first if and only if $U_{g}^{b}\geq U_{s}^{b}$, otherwise, the patient goes to the SP directly.

**Fig 1:**
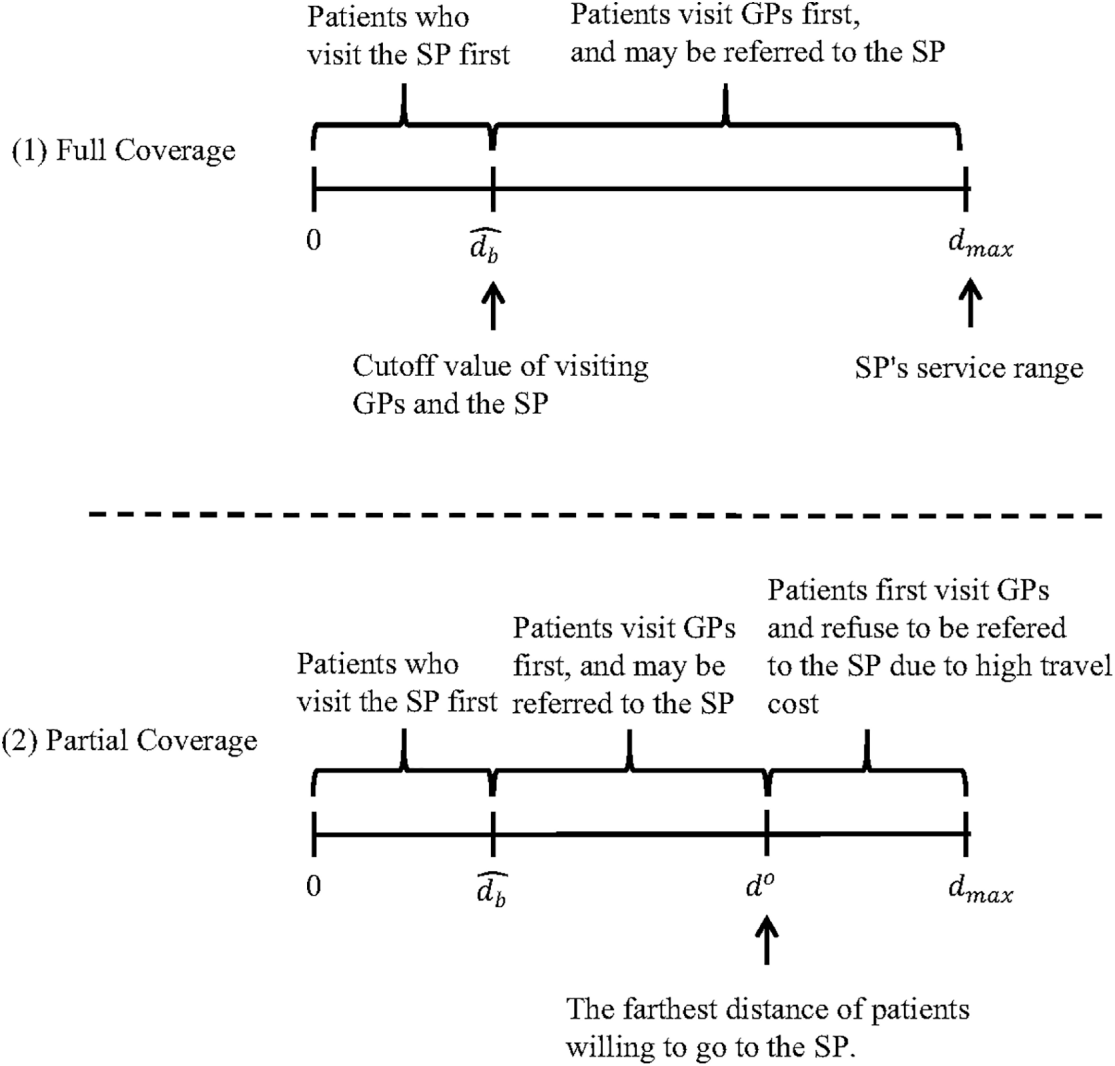
Market segmentation in the referral-based system without telemedicine.

As we have described above, top hospitals are better known than ordinary ones and can attract patients who live far away. Thus, we can assume the top hospital’s service range is larger than ordinary one. Based on SP’s maximum service range $d_{max}$, we consider two scenarios: full coverage and partial coverage. In full coverage setting, $d_{max}$ is small, that is, travel cost for patients within the service range is relatively small, then all patients have the opportunity to access the SP for treatment. While in partial coverage setting, $d_{max}$ is large and part of patients within the service range will give up treatment due to high travel cost (see Fig 1).

#### 3.1.1. Full coverage.

In this setting, as we mentioned before, a smaller $d_{max}$ implies the total number of patients in the system is small. All patients referred by GPs will go to the SP for further treatment when $U_{s}^{b}{|}_{d_{max}}=R_{s}-\left(\text{1}-q_{s}\right)P_{s}-QC\left(\lambda_{s}^{b}\right)-C_{t}d_{max}\geq \text{0}$ always satisfied.

When it reaches equilibrium, there is a $d={\hat{d}}_{b}$ makes $U_{g}^{b}=U_{s}^{b}$. The following lemma characterizes the patients’ decision given GPs’ referral rate, SP’s service rate, and other parameters (all proofs are in Appendix B in S1 Appendix).

**Lemma 1.** Cutoff value of visiting GPs and SP can be obtained by solving the following equation:


$${\hat{\;\;d}}_{b}=\frac{[m+(\text{1}-q_{g})P_{g}]k_{b}}{C_{t}F(k_{b})}+\frac{R_{s}-R_{g}-m-\left(\text{1}-q_{s}\right)P_{s}-QC\left(\lambda_{s}^{b}\right)}{C_{t}}$$ (3)

(1). Patients whose distance from SP $d\in [\text{0},{\hat{\;\;\;d}}_{b}]$ will choose to visit the SP directly; (2). Patients whose distance from SP $d\in (\;\;\;{\hat{\;\;d}}_{b},d_{max}]$ will choose to visit a GP first, and [1−F(k)] of which would be referred to the SP for further treatment.

Given the market segmentation, we can characterize the resulting demand in each service provider. For the SP, the provider determines the optimal service rate $\mu_{b}$ by maximizing his total profit, that is, maximizing his total arrival rate. We characterize the SP’s objective function as follow:


$$\underset{\mu_{b}}{\text{max}}\lambda_{s}^{b}={\hat{\;\;\;d}}_{b}+\left(d_{max}-{\hat{\;\;\;d}}_{b}\right)[\text{1}-F(k_{b})]$$

The first term represents the number of patients that choose to go to the SP for their first treatment, and the second term represents the number of patients that are referred by a GP, either directly refer or mistreatment. ${\hat{\;\;\;d}}_{b}$ represents the number of patients who go to the SP directly before the introduction of telemedicine.

For GPs, who need to make a referral decision to maximizing his profit, we formulate GPs’ profit function as following:


$$\underset{k_{b}}{\text{max}}\pi_{g}^{b}=\lambda_{g}^{b}\left\{\left(P_{g}-C_{g}\right)k_{b}-\text{m}\left[k_{b}-F\left(k_{b}\right)\right]\right\}$$ (4)

where $\lambda_{g}^{b}=\left(d_{max}-{\hat{\;\;\;d}}_{b}\right)$ is denoted as the total number of patients going to GP before the introduction of telemedicine, $C_{g}$ represents the GP’s treatment cost of per patient.

#### 3.1.2. Partial coverage.

Next, we consider the second scenario, where the service range of the SP $d_{max}$ is large, that is, part of patients within the service range will give up treatment due to high travel cost, i.e., $R_{s}-\left(\text{1}-q_{s}\right)P_{s}-QC\left(\lambda_{s}^{b}\right)-C_{t}d_{max}<\text{0}$.

Similar to the analysis of full coverage, we also calculate same cutoff value ${\hat{\;\;\;d}}_{b}$ to define the market segmentation. It is worth noting that unlike the case of full coverage, some patients will give up treatment because of the higher distance cost when $d_{max}$ is big enough. In this case, $R_{s}-\left(\text{1}-q_{s}\right)P_{s}-QC\left(\lambda_{s}^{b}\right)-C_{t}d^{o}=\text{0}$ where $d^{o}$ is the farthest distance of patients willing to visit the SP. We then give the following Lemma 2.

**Lemma 2.** The expression of ${\hat{d}}_{b}$ is identical to that in Lemma 1, and $d^{o}$ is as follow:


$$d^{o}=\frac{R_{s}-\left(\text{1}-q_{s}\right)P_{s}-QC\left(\lambda_{s}^{b}\right)}{C_{t}}$$ (5)

(1). Patients with $d\in [\text{0},{\hat{d}}_{b}]$ will choose to visit the SP directly; (2). Patients with $d\in ({\hat{d}}_{b},d^{o}]$ will choose to visit GPs first, and [1−F(k)] of which would be referred to the SP for further treatment; (3). Patients with $d\in (d^{o},d_{max}]$ will choose to visit GPs firstly, and give up further SP’s treatment due to high travel cost.

Similar to the above analysis, GPs’ objective function is the same as that of full coverage. For SP, the objective function is:


$$\underset{\mu_{b}}{\text{max}}\lambda_{s}^{b}={\hat{\;\;\;d}}_{b}+\left(d^{o}-{\hat{\;\;\;d}}_{b}\right)\left[\text{1}-F\left(k_{b}\right)\right]$$ (6)

### 3.2. After the introduction of telemedicine

After the introduction of SP’s telemedicine service, there are four choices for patients. A patient can (1) visits the SP in person directly; (2) visits the SP via telemedicine with the assistance of the GP; (3) visits a nearest GP first, and has [1−F(k)] probability of being referred to the SP in person; (4) visits a nearest GP first, and has [1−F(k)]probability of being referred to the SP via telemedicine. We use the following functions to describe the utility functions of patients’ different choices:

Patient’s expected utility Function at SP in person:


$$U_{s\text{1}}^{a}=R_{s}-\left(\text{1}-q_{s}\right)P_{s}-QC\left(\lambda_{s}^{a}\right)-C_{t}d$$ (7)

Expected utility Function of patients choosing telemedicine:


$$U_{s\text{2}}^{a}=R_{t}-\left(\text{1}-q_{t}\right)P_{s}-QC\left(\lambda_{s}^{a}\right)$$ (8)

where $\lambda_{s}^{a}$ is the total arrival rate at SP after the introduction of telemedicine.

Similar to the above analysis, we calculate the farthest distance of patients willing to go directly to SP in person after the introduction of telemedicine by $U_{s\text{1}}^{a}=U_{s\text{2}}^{a}$, that is, ${\tilde{\;\;\;d}}_{a}=\frac{R_{s}-R_{t}+\left(q_{i}-q_{t}\right)P_{s}}{C_{t}}$.

Then, for easily analysis, according to the above solution ${\tilde{\;\;\;d}}_{a}$, we analyze the expected utility function of patient when visiting a GP. When ${d_{i}\leq \tilde{\;\;\;d}}_{a}$, the patient i visits a GP firstly, and may be referred to the SP in person; otherwise, the patient i visits SP via telemedicine. Then we model the expected utility function of patient with different distance.

If patient i with ${d_{i}\leq \tilde{\;\;d}}_{a}$ goes to a GP first, she will visit the SP in person when she is referred. Expected utility Function of the patient can be formulated as:


$$U_{g\text{1}}^{a}=F\left(k_{a}\right)R_{g}-k_{a}\left(\text{1}-q_{g}\right)P_{g}-m\left[k_{a}-F\left(k_{a}\right)\right]+\left[\text{1}-F\left(k_{a}\right)\right]U_{s\text{1}}^{a}$$ (9)

Similarly, if patient i with ${d_{i}>\tilde{\;\;d}}_{a}$ goes to a GP first, patient i will visit the SP via telemedicine when she is referred. Expected utility Function of the patient can be formulated as:


$$U_{g\text{2}}^{a}=F\left(k_{a}\right)R_{g}-k_{a}\left(\text{1}-q_{g}\right)P_{g}-m\left[k_{a}-F\left(k_{a}\right)\right]+\left[\text{1}-F\left(k_{a}\right)\right]U_{s\text{2}}^{a}$$ (10)

Given GP’s referral rate $k_{a}$ and SP’s service rate $\mu_{a}$, patient i makes the visiting decision to maximize her expected utility, that is, $max\{U_{s\text{1}}^{a},U_{s\text{2}}^{a},U_{g\text{1}}^{a},U_{g\text{2}}^{a}\}$.

**Lemma 3.** Cutoff value of visiting GPs and SP can be obtained by solving the following equation:


$${\hat{\;\;d}}_{a}=\frac{[m+(\text{1}-q_{g})P_{g}]k_{a}}{C_{t}F(k_{a})}+\frac{R_{s}-R_{g}-m-\left(\text{1}-q_{s}\right)P_{s}-QC\left(\lambda_{s}^{a}\right)}{C_{t}}$$ (11)

and $\begin{array}{c} {\tilde{\;\;d}}_{a}=\frac{R_{s}-R_{t}+\left(q_{i}-q_{t}\right)P_{s}}{C_{t}} \end{array}$.

**Fig 2:**
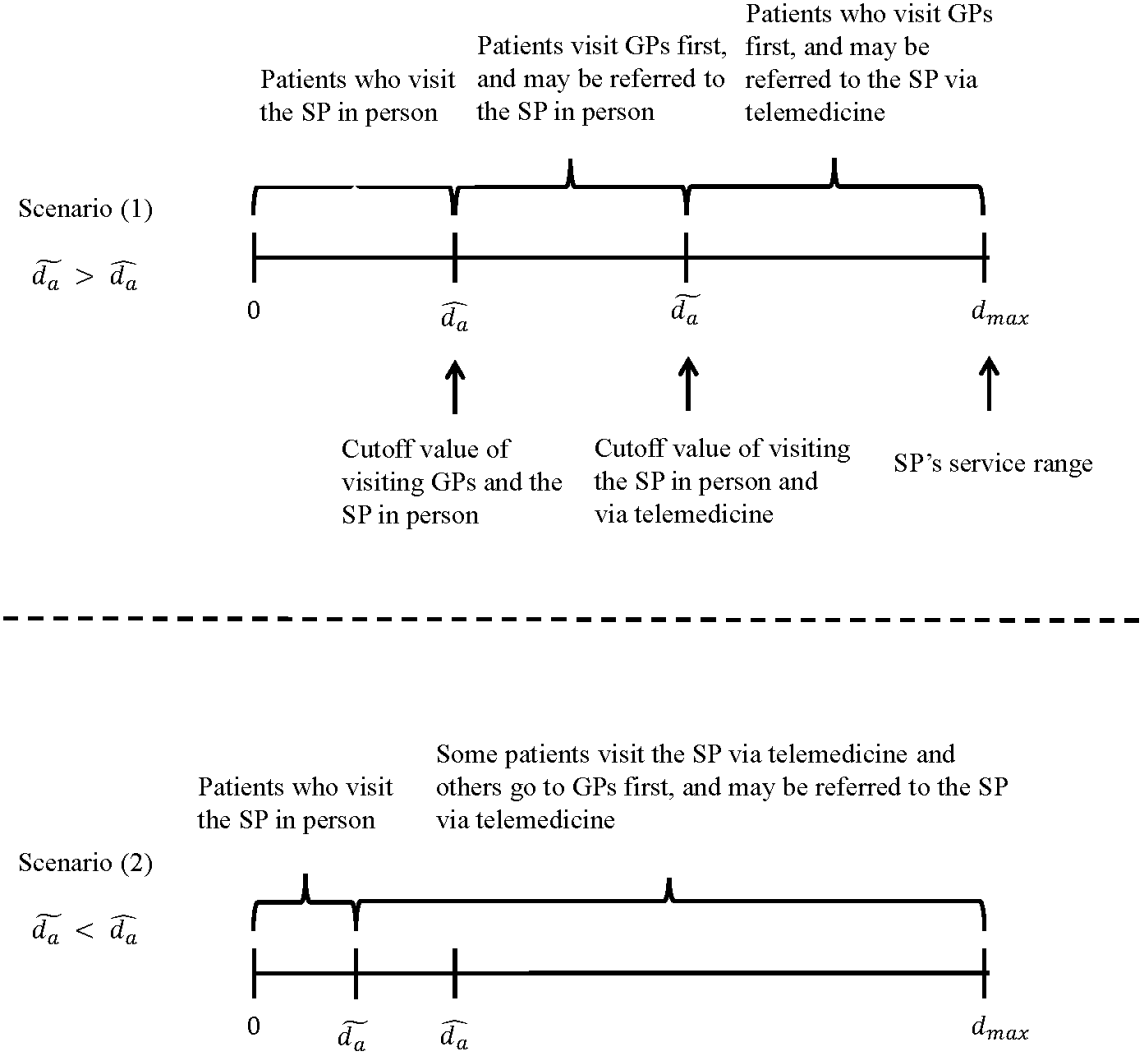
Market segmentation in the referral-based system with telemedicine.

For easily to analyze and compare patient’s decision, we consider two scenarios as follow (See Fig 2).

**Scenario 1:** ${\hat{\;\;\;d}}_{a}\leq \;{\tilde{\;\;d}}_{a}$

In this scenario, we can easily know that $U_{g\text{1}}^{a}=U_{s\text{1}}^{a}{|}_{{\hat{d}}_{a}}$ because ${\hat{\;\;\;d}}_{a}$ is the cutoff value of visiting GP and SP in person directly. Also, for the patient with ${\hat{\;\;\;d}}_{a}$, the expected utility of visiting the SP in person is bigger than with telemedicine, that is, $U_{s\text{1}}^{a}{|}_{{\hat{d}}_{a}}>U_{s\text{2}}^{a}$. Then, we have $U_{g\text{1}}^{a}>U_{s\text{2}}^{a}$, which represent that none of patients choose telemedicine for their first visit in this scenario.

We have (1) For any patients with $d\in [\text{0},{\hat{\;\;\;d}}_{a}]$, they will choose to visit the SP directly; (2) For any patients with $d\in (\;{\hat{\;\;d}}_{a},{\tilde{\;\;d}}_{a}]$, they will choose to visit GPs first and if necessary, they will be referred to the SP in person; (3) For any patients with $d\in ({\tilde{\;\;d}}_{a},d_{max}]$, they will choose to visit GPs first and if necessary, they will be referred to the SP via telemedicine.

We then formulate the SP’s objective function, which is equivalent to:


$$\underset{\mu_{a}}{\text{max}}\lambda_{s}^{a}=d_{max}-\left(d_{max}-{\hat{\;\;\;d}}_{a}\right)F\left(k_{a}\right)$$ (12)

Also, GPs’ objective function:


$$\underset{k_{a}}{\text{max}}\pi_{g}^{a}=\left(d_{max}-{\hat{\;\;\;d}}_{a}\right)\left\{\left(P_{g}-C_{g}\right)k_{a}-m\left[k_{a}-F\left(k_{a}\right)\right]\right\}+\gamma \left(d_{max}-\tilde{\;\;d}\;\right)\left[\text{1}-F\left(k_{a}\right)\right]$$ (13)

**Scenario 2:** ${\hat{\;\;\;d}}_{a}>{\tilde{\;\;d}}_{a}$

Different with Scenario 1, some patients will choose to visit the SP via telemedicine when $d>{\tilde{\;\;d}}_{a}$. That is, there exist ${U_{g\text{1}}^{a}=U}_{s\text{1}}^{a}{|}_{{\tilde{d}}_{a}}>U_{s\text{2}}^{a}{|}_{{\hat{d}}_{a}}$ when ${\hat{\;\;\;d}}_{a}>{\tilde{\;\;d}}_{a}$. We denote $\lambda_{tele}$ as the number of patients choosing telemedicine for their first visit.

We have (1). For any $d\in [\text{0},{\tilde{\;\;d}}_{a}]$, a patient will choose to visit the SP directly; (2). For some patients with $d\in (\;\;{\tilde{\;\;d}}_{a},d_{max}]$, $\lambda_{tele}$ patients prefer telemedicine for her first visit, and other patients with $d\in (\;\;{\tilde{\;\;d}}_{a},d_{max}]$ will choose to visit GPs first and if necessary, they will be referred to the SP via telemedicine.

Similar to Scenario 1, SP’s objective function is equivalent to:


$$\underset{\mu_{a}}{\text{max}}\lambda_{s}^{a}=d_{max}-\left(d_{max}-\;\;{\tilde{\;\;d}}_{a}-\lambda_{tele}\right)F\left(k_{a}\right)$$ (14)

GP’s objective function:


$$\underset{k_{a}}{\text{max}}\pi_{g}^{a}=\left(d_{max}-\;\;{\tilde{\;\;d}}_{a}-\lambda_{tele}\right)\left\{\left(P_{g}-C_{g}\right)k_{a}-\text{m}\left[k_{a}-F\left(k_{a}\right)\right]\right\}$$

$$+\gamma \left\{\lambda_{tele}+\left(d_{max}-\;\;{\tilde{\;\;d}}_{a}-\lambda_{tele}\right)\left[\text{1}-F\left(k_{a}\right)\right]\right\}$$ (15)

## 4. Analysis results

### 4.1. Optimal solutions of GPs and SP

As our model involves multiple variables, it is not easy to analytically identify the unique optimal solution for GP’s profit function. Many researchers have used numerical analysis to verify that the profit function is unimodal and there exists a unique optimal solution. For example, Luo et al (2017) used the numerical analysis methods to solve the problem given that the optimal analytical solution to the profit function is unattainable [26]. Thus, we numerically show the existence of the best solution.

**Fig 3:**
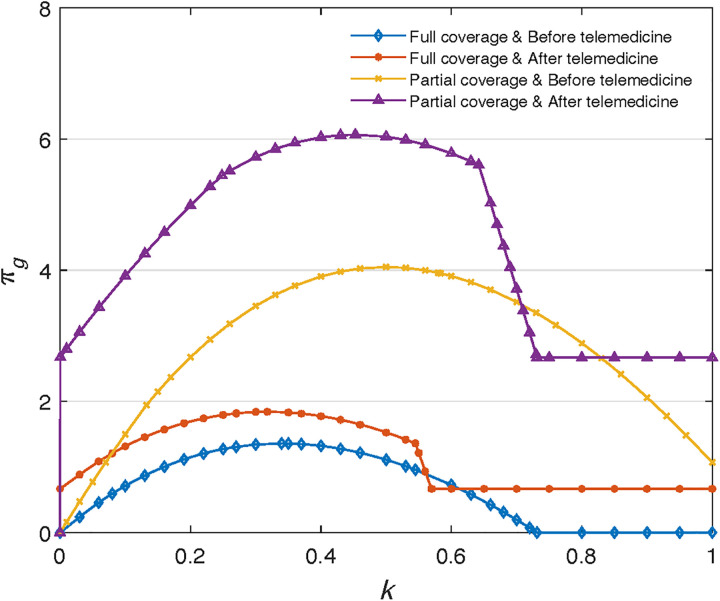
The existence of GPs’ optimal solution.

As shown in Fig 3, the GP’s profit function exhibits a unimodal shape, confirming the existence of an optimal referral level. The peak of the curve, corresponds to the referral rate that maximizes the GP’s profit. This optimal point reflects the trade-off faced by the GP between treating patients directly and referring them to the SP, taking into account both treatment cost and mistreatment cost. Identifying this optimal referral threshold is essential for understanding how financial incentives and operational constraints shape GP behavior in the referral system.

**Proposition 1.** Before and after the introduction of telemedicine, based on GPs’ referral decision $k_{j},j=\{b,a\}$, SP’s response function is:


$$\frac{\beta }{{\left(\mu -\lambda_{s}^{j}\right)}^{\text{2}}}=\alpha j=\left\{b,a\right\}$$ (16)

Proposition 1 suggests that SP would optimize his service quality to attract more patients to go to SP directly to maximize his profit. According to the equation above, we can also figure out patients waiting time $\frac{\text{1}}{\mu -\lambda_{s}}$ is constant no matter what patients’ arrival rate at SP is. However, more patients would lead to a higher service rate. Thus, the SP’s service quality would decrease when he has more patients.

### 4.2. Numerical study

In this section, we use numerical experiments to explore the impact of telemedicine in a referral-based healthcare system with heterogeneous consumers. We specifically examine how the GP’s tele-reward (γ) and patient’s travel cost ($C_{t}$) influence key decisions and outcomes, including the GP’s referral threshold (k), SP service rate (μ), average quality cost (ϕ), and total social welfare (TSW).

Considering the difficulty of data acquisition and some unquantifiable costs, as well as the generality of a set of real data to all regions, we just capture the relationship between variables to define the range of parameters.

Our study included a wide ranging set of parameter values so as to ensure that we consider a broad range of referral threshold k values found in the real world is from 7% to 77% [27]. There is no public source for mistreatment cost m and so we considered a good surrogate for it which is the malpractice insurance cost. The average malpractice insurance cost per year for internal medicine ranges from $8000 to $47,000 [28]. In addition, the average panel size of a GP is 2300 patients [29]. Using these two numbers, we deduce that mistreatment cost per patient is in the range 3.5 to 20.5. To focus on cases where we have interior solutions, we considered the following set of values: m∈[10,15]. Next, SP incomes are typically about 2–3 times that of GPs [30] and so, based on the values of m,k, we chose the following ranges for the parameters: $P_{g}\in [\text{1}\text{0},\text{1}\text{5}],C_{g}\in [\text{4},\text{6}],P_{s}\in [\text{1}\text{5},\text{2}\text{0}],C_{s}\in [\text{6},\text{8}],\gamma \in [\text{2},\text{4}]$.

We now discuss the approach used to estimate the treatment failure cost α at the SP. In a prior study, Adida and Bravo (2019) estimated this cost to range from 0.3 to 1.1 times the treatment cost at SP [31]. The treatment cost represents the average SP cost per patient, which ranges from 6 to 8 (range of $C_{s}$ values) when service rate μ=1. Therefore, the α values were picked from the following interval: α∈[2,4]. There is no commonly available source for the cost of delayed care per unit delay [32]. So, based on our assumption and the range of other parameters, we found that the values of β should be in the range: β∈[2,4]. As for other parameters, we have mentioned before $R_{s}>R_{t}>R_{g}$ and $q_{g}>q_{t}>q_{s}$. Based on patients’ utility function and costs incurred in the process of treatment. We set $R_{g}=\text{10},R_{s}=\text{2}\text{0},R_{t}=\text{1}\text{5},q_{g}=\text{0}.\text{9},q_{s}=\text{0}.\text{8},q_{t}=\text{0}.\text{8}\text{5}$. And travel cost per unit distance $C_{t}$ were picked from the following interval: $C_{t}\in [\text{6},\text{8}]$.

We compare the performance of telemedicine under full coverage and partial coverage. We also focus on the effect of GP’s tele-reward (γ) and patient’s travel cost $\left(C_{t}\right)$ on optimal GP’s referral rate (k), SP’s service rate (μ), average quality cost (ϕ), and total social welfare (TSW) under all cases. Average quality cost is defined as the quality cost of curing a patient, i.e., total quality cost/the number of cured patients. Total quality cost include mistreatment cost incurred at GPs $\lambda_{g}m[k-F(k)]$ and quality cost incurred at SP $\lambda_{s}QC(\lambda_{s})$. Total social welfare refers to total patients’ reward from seeking healthcare service minus all costs.

**Fig 4:**
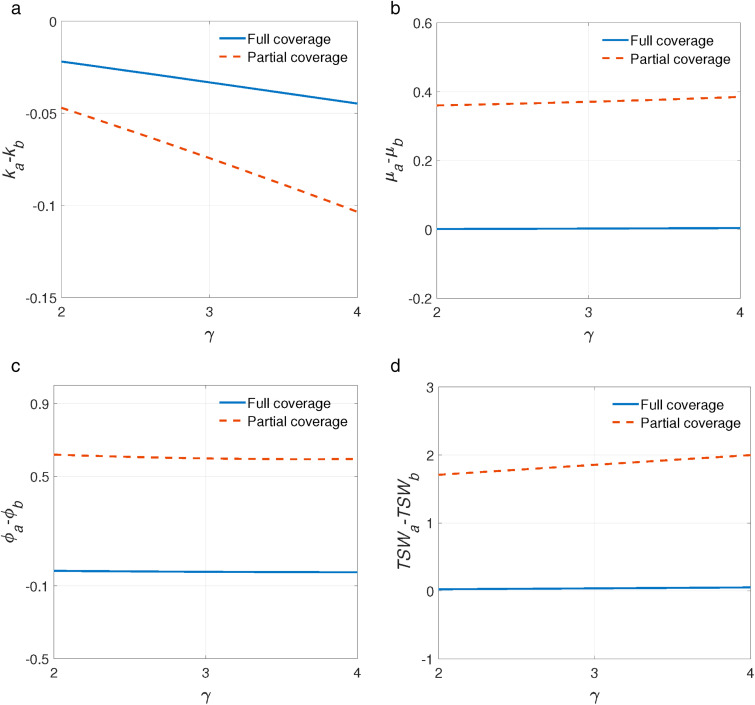
The impact of tele-reward γ on the performance of telemedicine. a. $k_{a}-k_{b}\text{vs}.\gamma$. b. $\mu_{a}-\mu_{b}\text{vs}.\gamma$. c. $\phi_{a}-\phi_{b}\text{vs}.\gamma$. d. ${TSW}_{a}-{TSW}_{b}\text{vs}.\gamma$.

**Fig 5:**
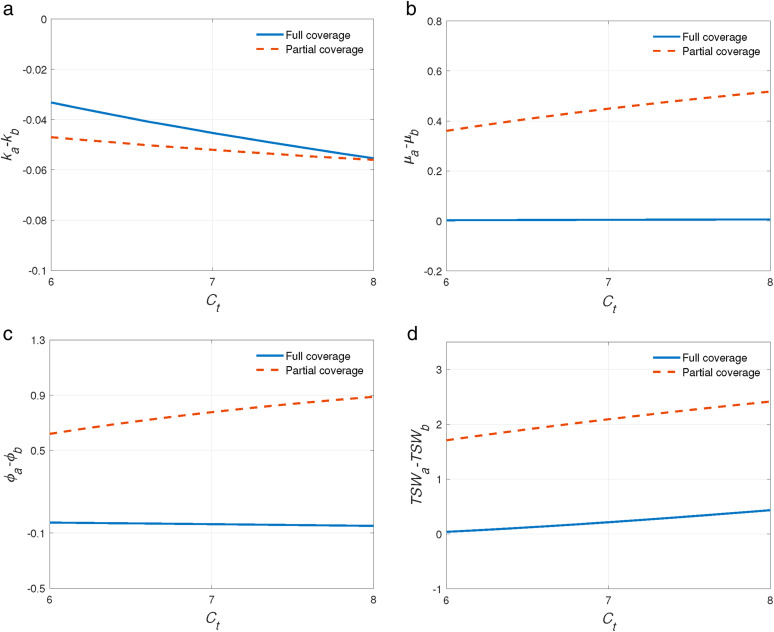
The impact of travel cost $C_{t}$ on the performance of telemedicine. a. $k_{a}-k_{b}\text{vs}.C_{t}$. b. $\mu_{a}-\mu_{b}\text{vs}.C_{t}$. c. $\phi_{a}-\phi_{b}\text{vs}.C_{t}$. d. ${TSW}_{a}-{TSW}_{b}\text{vs}.C_{t}$.

As shown in Figs 4 and 5, we have after the introduction of telemedicine, GPs would decrease their referral level k since GPs could gain tele-reward by assisting in the telemedicine. SP would increase his service rate due to the increase in patients’ arrival rate (GPs refers more patients to him). It’s interesting that average quality cost ϕ would decrease after the introduction of telemedicine under full coverage while increase under partial coverage as a result of excessive load of SP. When there are fewer patients in the system (full coverage), GPs refer more patients to SP that could decrease their mistreatment cost. However, under partial coverage, highly utilized specialist could become substantial and consequently lead to lower service quality. With the application of telemedicine, some patients could choose telemedicine instead of going to the SP in person so that they can save travel costs. Thus, TSW would increase. It is worth noting that telemedicine makes more patients access to treatment under partial coverage, therefore, the impact of telemedicine on TSW is more significant under partial coverage than under full coverage.

It’s obvious to see that the impacts of the introduction of telemedicine on the referral level k, service rate μ, average quality cost ϕ and total social welfare TSW are more significant under partial coverage. In addition, telemedicine has a greater impact with the increase of γ and $C_{t}$.

Next, we use partial coverage as the example to explore the impact of tele-reward γ and travel cost $C_{t}$ in referral-based healthcare system since these two variables are the most relevant variables in the analysis of telemedicine. The impact of telemedicine under full coverage has similar results to the analysis of partial coverage and is omitted.

**Fig 6:**
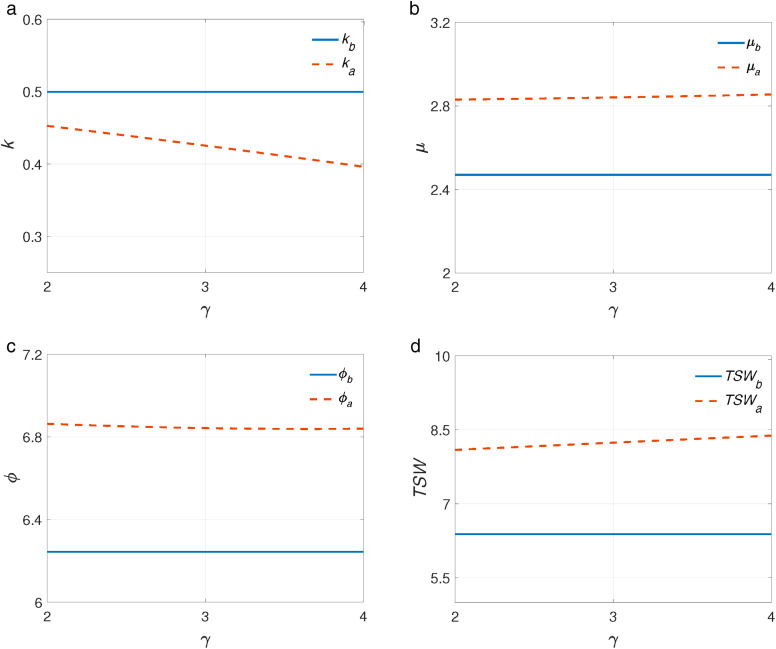
The impact of tele-reward γ under partial coverage. a. kvs.γ. b. μvs.γ. c. ϕvs.γ. d. TSWvs.γ.

As shown in Fig 6, after the introduction of telemedicine, referral level k slightly decreases in γ. The intuitive is that GPs are more willing to referral patients to SP via telemedicine with the increase of γ, which not only increase GPs’ profit but also decrease mistreatment cost (see Fig 6a). SP’s service rate increases in γ, this phenomenon is related to GPs’ response according to Proposition 1. That is, the number of patients is referred by GPs increases (see Fig 6b). Fig 6c and Fig 6d show that the average quality cost ϕ decreases and total social welfare TSW increases in γ. This is because higher tele-reward incentivizes GPs to refer more patients to the SP which could help resolve excessive mistreatment. It also indicates that the incentives to GPs could boost the effectiveness of telemedicine.

**Fig 7:**
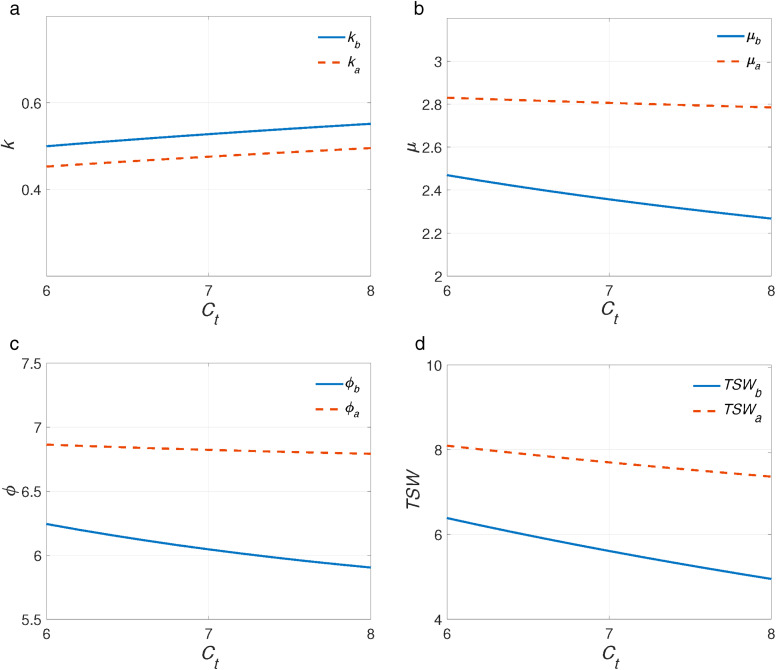
The impact of travel cost $C_{t}$ under partial coverage. a. $k\text{vs}.C_{t}$. b. $\mu \text{vs}.C_{t}$. c. $\phi \text{vs}.C_{t}$. d. $TSW\text{vs}.C_{t}$.

With the increase of $C_{t}$, patients are less willing to visit the SP in person due to higher travel cost. Then, more people choose to visit GPs first, in this case, GPs increase their referral rate due to higher mistreatment cost to respond to patients’ choice behavior (see Fig 7a). Also, service rate of the SP μ decreases due to the decreased arrival rate (see Fig 7b). We also found that more patients likely to visit the GPs that would lighten the load of highly utilized SP and therefore improve the system’s cost-effectiveness (see Fig 7c). However, despite the operational benefits, higher travel costs still lead to a decline in patients welfare, particularly for those in remote areas (see Fig 7d).

To further verify the robustness of our findings, we conducted additional sensitivity analyses by varying other key parameters such as the mistreatment cost at GP (m), delayed care cost at SP (β), SP’s service cost ($C_{s}$), and cost of inadequate care at SP (α). These parameters were adjusted within empirically reasonable ranges based on prior literature and practice-oriented estimates. The results show that although these parameter variations slightly affect the numerical values of outcomes, the qualitative patterns and key insights remain unchanged.

Due to the minimal variation observed and to maintain the clarity and conciseness of the manuscript, we chose not to present additional figures for these robustness checks. All omitted figures can be provided upon request.

## 5. Discussion and conclusion

This work has developed analytical models for describing the patients’ healthcare-seeking decisions and the GPs’ referral decisions in different scenarios of service coverage. We conduct numerical experiments to examine how the introduction of telemedicine impacts the collaboration between the service providers and the speed-quality trade-off inside the healthcare delivery system, and how tele-reward to GPs γ and travel cost $C_{t}$ incentivizes the patients and the providers to make decisions. Our analysis has yielded a number of interesting insights.

Our results reveal a clear difference across coverage settings. In full coverage systems, where the SP is underutilized, the introduction of telemedicine improves accessibility without overloading the SP, resulting in lower mistreatment costs and better overall quality. By contrast, in partial coverage systems, telemedicine significantly improves social welfare by allowing more patients who would otherwise forgo treatment (due to high travel cost) to receive care remotely. Thus, telemedicine is more significant in improving social welfare in partial coverage scenarios. However, this benefit comes at the cost of a heavier patient inflow to the SP through GP referrals. Given fixed capacity, the SP is forced to increase his service rate, which leads to longer queues and shorter consultation times, and ultimately causes a decline in service quality. This finding is driven by a dual mechanism: telemedicine simultaneously expands access and intensifies capacity utilization. This highlights the important role of capacity planning and resource allocation when implementing telemedicine in overburdened healthcare systems.

Our numerical experiments further highlight the role of tele-reward (γ) and travel cost per unit distance ($C_{t}$) as two key moderating factors that influence the effectiveness of telemedicine. Under partial coverage scenarios, we find that an increase in γ incentivizes GPs to refer more patients via telemedicine, reducing the likelihood of mistreatment and improving both GP profits and overall system performance. The increased referrals drive up the SP’s service rate, which may raise concerns over service quality degradation; however, the net impact on average quality cost is mitigated due to the reduced misdiagnosis at the GP level. Consequently, TSW increases significantly with higher γ, suggesting that appropriate financial incentives can substantially boost telemedicine’s operational efficiency.

In contrast, when travel cost $C_{t}$ increases, patients become less willing to travel to SP for in-person care, making GPs the primary point of access. This leads GPs to adopt more cautious referral strategies and increases the uptake of telemedicine. The reduced patient flow to the SP alleviates congestion and, paradoxically, improves average service quality at the SP. However, the overall utility of patients may still decline due to the burden of high travel costs, especially for those in underserved areas, indicating that accessibility alone is not sufficient without addressing affordability and system responsiveness.

The above discussion not only demonstrates the significant impact of telemedicine on service delivery in a referral-based healthcare system, but also highlights the influence factors that can impact its effectiveness. These findings yield important insights into the economic feasibility of telemedicine and its broader policy implications. To ensure that the expansion of telemedicine does not compromise service quality, initiatives should be accompanied by rigorous capacity planning and outcome monitoring. Policy priorities should further adapt to the degree of regional healthcare coverage: in under-served areas, telemedicine can be strategically scaled to alleviate access disparities, whereas in well-covered systems, emphasis should shift toward enhancing operational efficiency and managing complex case flows. Financial incentive mechanisms for general practitioners (GPs) should be designed with contextual sensitivity. In remote or high-demand regions, stronger incentive parameters may promote greater GP engagement in tele-coordination, thereby improving triage efficiency and referral appropriateness. In areas where travel costs remain prohibitive, policy efforts should prioritize investments in broadband connectivity, interoperable digital platforms, and patient-side support measures to ensure equitable access and sustained participation in telemedicine.

Overall, telemedicine should be treated as a system-sensitive intervention, rather than a universally beneficial tool. The trade-off between expanded access and quality preservation must be carefully managed through integrated health planning and targeted tele-reward designs.

## Supporting information

S1 Appendix Notation and Proofs. (DOCX)

S1 File Experimental results. (XLSX)
