# Supplementary material for: Can telemedicine help integrate the referral-based healthcare system? An economic and operational analysis
Source: PLoS One. 2025 Nov 14;20(11):e0336490. doi: 10.1371/journal.pone.0336490 (PMC12617913; doi:10.1371/journal.pone.0336490)
Supplement: S1 Appendix — (DOCX) [file pone.0336490.s001.docx]

**Appendix A: Notation**

| **Variables** | |
| --- | --- |
| $k$ | Complexity threshold (GPs treat all patients with complexity $x\leq k$) |
| $f(x)$ | Probability of GP successfully treats a patient with complexity $x$ |
| $F(x)$ | Proportion of patients successfully treated by GP, given $k$ ($\equiv\int_{0}^{k} f(x)dx$) |
| $\lambda_{g}$ | Arrival rate at GPs |
| $\lambda_{tele}$ | The number of patients choosing telemedicine for their first visit |
| $\lambda_{s}$ | Arrival rate at SP |
| $\mu$ | Service rate at SP |
| $QC(\lambda_{s})$ | A patient’s service quality cost incurred at the SP |
| $W({\mu, \lambda}_{s})$ | Waiting time for SP, given $\lambda_{s}$ and $\mu$ |
| $\phi$ | Average service quality cost |
| TSW | Total social welfare |
| **Parameters** | |
| $R_{g}$ | The reward of a patient when she visits a GP |
| $R_{s}$ | The reward of a patient when she visits the ED |
| $R_{t}$ | The reward of a patient when she is treated by the SP via telemedicine |
| $P_{g}$ | Treatment price of GPs per patient |
| $P_{s}$ | Treatment price of SP per patient |
| $q_{g}$ | The reimbursement rate of GPs |
| $q_{s}$ | The reimbursement rate of SP in person |
| $q_{t}$ | The reimbursement rate of telemedicine |
| $C_{g}$ | Service Cost of GP per patient |
| $C_{s}$ | Service Cost of ED per unit time |
| $m$ | Treatment failure cost at GP per patient |
| $\beta$ | Delayed care (Waiting) cost at SP per patient per unit waiting time |
| $\alpha$ | cost of inadequate care at SP |
| $\gamma$ | Tele-reward to a GP when he assists in telemedicine |
| $C_{t}$ | Travel cost per unit distance |

**Appendix B: Proofs**

**Proof of Lemma 1**

When it reaches equilibrium, there is a $d=\hat{d}_{b}$ makes $U_{g}^{b}=U_{s}^{b}$. Based on equations 1 and 2, we have $R_{s}-\left( 1-q_{s} \right)P_{s}-QC\left( \lambda_{s}^{b} \right)-C_{t}\hat{d}_{b}=F\left( k_{b} \right)R_{g}-k_{b}\left( 1-q_{g} \right)P_{g}-m\left[ k_{b}-F\left( k_{b} \right) \right]+[1-F(k_{b})][R_{s}-\left( 1-q_{s} \right)P_{s}-QC\left( \lambda_{s}^{b} \right)-C_{t}\hat{d}_{b}]$*.* After simplification, it is easy to derive that $\hat{d}_{b}=\frac{[m+\left( 1-q_{g} \right)P_{g}]k_{b}}{C_{t}F\left( k_{b} \right)}+\frac{R_{s}-R_{g}-m-\left( 1-q_{s} \right)P_{s}-QC\left( \lambda_{s}^{b} \right)}{C_{t}}$.

**Proof of Lemma 2**

When patients’ distance from the SP is high enough, they will give up treatment because of the high travel cost. In this case, the farthest distance of patients will to visit the SP, $d^{o}$, needs to satisfy $R_{s}-\left( 1-q_{s} \right)P_{s}-QC\left( \lambda_{s}^{b} \right)-C_{t}d^{o}=0$. Thus, we have $d^{o}=\frac{R_{s}-\left( 1-q_{s} \right)P_{s}-QC\left( \lambda_{s}^{b} \right)}{C_{t}}$.

**Proof of Lemma 3**

Analogous to the analysis before the introduction of telemedicine, we have $\hat{d}_{a}=\frac{[m+(1-q_{g})P_{g}]k_{a}}{C_{t}F(k_{a})}+\frac{R_{s}-R_{g}-m-\left( 1-q_{s} \right)P_{s}-QC\left( \lambda_{s}^{a} \right)}{C_{t}}$.

The farthest distance of patients willing to go directly to SP in person after the introduction of telemedicine, $\tilde{d}_{a}$, can be calculated by solve $U_{s1}^{a}=U_{s2}^{a}$ as shown in equations 7 and 8. So we have $R_{s}-\left( 1-q_{s} \right)P_{s}-QC\left( \lambda_{s}^{a} \right)-C_{t}\tilde{d}_{a}=R_{t}-\left( 1-q_{t} \right)P_{s}-QC\left( \lambda_{s}^{a} \right)$. After simplification, it is easily to derive that $\tilde{d}_{a}=\frac{R_{s}-R_{t}+\left( q_{i}-q_{t} \right)P_{s}}{C_{t}}$.

**Proof of Proposition 1**

In the cases before the introduction of telemedicine and scenario (1) after the introduction of telemedicine, given any GPs' referral level $k$, SP's arrival rate $\lambda_{s}=\hat{d}+\left[ 1-F\left( k \right) \right][\min\left( d_{max}, d^{o} \right)-\hat{d}]$ and we also know $\frac{\partial\lambda_{s}}{\partial\hat{d}}=F(k)$. Based on Assumption 1, if the SP wants to maximize the arrival rate, he would attract as many patients as possible to visit him in person, i.e., maximize $\hat{d}$. Since $\hat{d}=\frac{[m+(1-q_{g})P_{g}]k}{C_{t}F(k)}+\frac{R_{s}-R_{g}-m-\left( 1-q_{s} \right)P_{s}-QC\left( \lambda_{s} \right)}{C_{t}}$ based on Lemma 1 and Lemma 3, we have $\frac{\partial\hat{d}}{\partial\mu}=-\frac{1}{C_{t}}[-\frac{\beta}{\left( \mu-\lambda_{s} \right)^{2}}\left( 1-F\left( k \right)\frac{\partial\hat{d}}{\partial\mu} \right)+\alpha]$. After simplification and transforming, we have $\frac{\partial\hat{d}}{\partial\mu}=\frac{\frac{\beta}{\left( \mu-\lambda_{s} \right)^{2}}-\alpha}{C_{t}+\frac{F(k)\beta}{\left( \mu-\lambda_{s} \right)^{2}}}$. It is easy to know that $\hat{d}$ is a unimodal function of $\mu$ that increases at first and then decreases. Thus, we have SP’s response function is $\frac{\beta}{{(\mu-\lambda_{s})}^{2}}=\alpha$ which suggests that SP would optimize his service quality.

In the case of scenario (2) after the introduction of telemedicine, SP would attract more patients to choose telemedicine through optimize his service quality since $\tilde{d}_{a}$ is constant. Analogous to the analysis above, we would derive the same conclusion.

Hence, we have SP's response function is: $\frac{\beta}{{(\mu-\lambda_{s})}^{2}}=\alpha$.
